# What Additional Factors Beyond State-of-the-Art Analytical Methods Are Needed for Optimal Generation and Interpretation of Biomonitoring Data?

**DOI:** 10.1289/ehp.0901108

**Published:** 2009-06-24

**Authors:** Antonia M. Calafat, Larry L. Needham

**Affiliations:** Division of Laboratory Sciences, National Center for Environmental Health, Centers for Disease Control and Prevention, Atlanta, Georgia, USA

**Keywords:** BPA, contamination, DEHP, extraction efficiency, field blank, phthalates

## Abstract

**Background:**

The routine use of biomonitoring (i.e., measurement of environmental chemicals, their metabolites, or specific reaction products in human biological specimens) to assess internal exposure (i.e., body burden) has gained importance in exposure assessment.

**Objectives:**

Selection and validation of biomarkers of exposure are critical factors in interpreting biomonitoring data. Moreover, the strong relation between quality of the analytical methods used for biomonitoring and quality of the resulting data is well understood. However, the relevance of collecting, storing, processing, and transporting the samples to the laboratory to the overall biomonitoring process has received limited attention, especially for organic chemicals.

**Discussion:**

We present examples to illustrate potential sources of unintended contamination of the biological specimen during collection or processing procedures. The examples also highlight the importance of ensuring that the biological specimen analyzed both represents the sample collected for biomonitoring purposes and reflects the exposure of interest.

**Conclusions:**

Besides using high-quality analytical methods and good laboratory practices for biomonitoring, evaluation of the collection and handling of biological samples should be emphasized, because these procedures can affect the samples integrity and representativeness. Biomonitoring programs would be strengthened with the inclusion of field blanks.

## Interpretation of Biomonitoring Data: Do Collection Protocols Matter?

Biomonitoring (i.e., measurement of environmental chemicals, their metabolites, or specific reaction products in human biological specimens) to assess internal exposure (i.e., body burden) has increased considerably in the last two decades (Needham et al. 2007). Biological matrices are complex; some may be difficult to obtain and available only in small amounts. Moreover, environmental chemicals are normally present in the biological matrix at trace levels. Therefore, highly sensitive, specific, and selective multianalyte methods for the extraction, separation, and quantification of these chemicals must be developed (Needham et al. 2005). Undoubtedly, the adequacy of biomonitoring data depends strongly on reliable analytical measurements (Angerer et al. 2007). Even when the best techniques are used, they guarantee accurate and precise measures of the biomarkers levels only in any given specimen. However, if the integrity of the specimen was compromised before its analysis, the analytical measures, although valid, could lead to erroneous interpretations. Sampling, storage, and processing conditions have long been appreciated as potential sources of contamination in trace analyses for metals and volatile organic compounds (Ashley et al. 1992; Bolann et al. 2007; Pineau et al. 1993). Unfortunately, adequacy of sampling and processing methods, albeit critical for the evaluation of all biomonitoring data, has not received as much attention as the analytical techniques, especially for semivolatile organic chemicals.

Strict collection, handling, and storage protocols are particularly important if the chemicals monitored as exposure biomarkers are ubiquitous environmental contaminants or environmental degradates. Some of these chemicals [e.g., phthalates, polybrominated diphenyl ethers, polyfluoroalkyl chemicals, bisphenol A (BPA)] have been detected in indoor air and dust (Fromme et al. 2009; Hwang et al. 2008; Rudel and Perovich 2009; Volkel et al. 2008; Weschler 2009; Wilson et al. 2003). Therefore, care must be taken when collecting and processing specimens to be analyzed for these chemicals to ensure that sampling materials do not contain detectable levels of the target chemicals, but also that these materials are dust-free. Further contamination with such chemicals during the analysis is possible. However, laboratory contamination, should it occur, would be identified and eliminated, provided that the laboratory performing the analysis adheres to good laboratory practices and includes analytical/reagent blank samples (Taylor 1987). Therefore, the resulting biomarker concentrations should never include a contribution from contamination during laboratory analyses.

Just as reagent blanks are needed for assessing contamination during the analytical steps, field blanks can be used to assess potential contamination during sample collection, storage, processing, and/or transport [National Institute for Occupational Safety and Health (NIOSH) 1994]. However, to further complicate matters, even if field blanks are used, additional information may be needed to determine the utility of the biomonitoring findings. In this article, we present examples that highlight the relevance of unforeseen and unintended contamination before laboratory analysis and its impact on the interpretation of biomonitoring data of organic chemicals. We also discuss the representativeness of specimens to be used for biomonitoring purposes. Other factors important for ensuring the adequate interpretation of biomonitoring results, including the selection of the most relevant biomarkers—based on available toxicokinetic data—for the chemical and population of interest; the potential effects of the biological matrix on the biomarkers’ concentrations (e.g., matrix enzymes and levels of some phthalate metabolites); and adequate storage and shipment of specimens (Angerer et al. 2007; Calafat and Needham 2008; National Research Council 2006) are not discussed.

## Potential Contamination during Sampling or Handling of Biological Specimens

**Figure 1:**
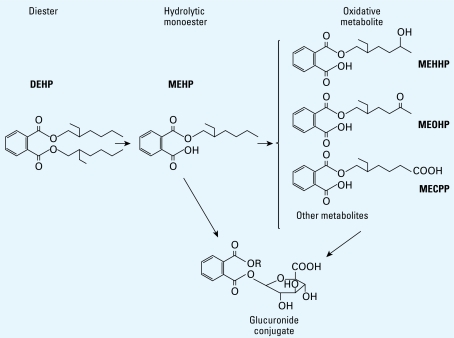
DEHP metabolizes into its hydrolytic monoacid (“monoester”) MEHP and, after enzymatic oxidation of the alkyl chain (R), to various oxidative metabolites. MEHP and the oxidative metabolites can be excreted in the urine unchanged or as phase II glucuronide conjugates [R = CH_2_CH(C_2_H_5_)(CH_2_)_3_CH_3_ (MEHP); CH_2_CH(C_2_H_5_)(CH_2_)_2_CH(OH)CH_3_ (MEHHP); CH_2_CH(C_2_H_5_)(CH_2_)_2_COCH_3_ (MEOHP); CH_2_CH(C_2_H_5_) (CH_2_)_3_COOH [mono(2-ethyl-5-carboxypentyl) phthalate (MECPP)].

For chemicals that are ubiquitous in the environment, such as certain phthalates, care is needed to avoid contaminating the samples. For example, contamination of biological specimens with di(2-ethylhexyl) phthalate (DEHP), a common plasticizer of polyvinyl chloride (PVC) and other polymers (David et al. 2001) used in many products, is difficult to avoid. In humans, DEHP metabolizes into its hydrolytic monoacid (commonly referred as “monoester”), mono(2-ethylhexyl) phthalate (MEHP), and then into oxidative metabolites (Figure 1) (Koch et al. 2004, 2005b, 2006; Silva et al. 2006). MEHP and the oxidative metabolites are primarily excreted in the urine as phase II conjugates and much less so as the unchanged or free species (Dirven et al. 1993; Peck and Albro 1982). Measuring the urinary concentrations of the total (conjugated plus free) species of these metabolites is the most common biomonitoring approach for assessing human exposure to DEHP (Barr et al. 2003; Koch et al. 2003).

To evaluate the exposure to several contaminants, including the metabolites of DEHP and other phthalates, among participants of the Avon Longitudinal Study of Parents and Children (ALSPAC) (Golding et al. 2001), we analyzed one pooled urine sample, prepared from 20 individual specimens, for these contaminants (Holmes et al., in press). Although in a given urine specimen, the concentrations of the total species of DEHP oxidative metabolites are normally higher than the total MEHP concentrations (Barr et al. 2003; Koch et al. 2003), in the ALSPAC pooled sample, the total urinary concentration of MEHP (100 μg/L) was one order of magnitude higher than the total concentrations of DEHP oxidative metabolites mono(2-ethyl-5-hydroxyhexyl) phthalate (MEHHP; 13.8 μg/L) and mono(2-ethyl-5-oxohexyl) phthalate (MEOHP; 12.7 μg/L). Furthermore, the MEHP concentrations were about 25 times higher than the median concentrations reported for the general population of the U.S. National Health and Nutrition Examination Survey (NHANES) conducted during 2001–2002, but the concentrations of MEHHP and MEOHP were very similar to the concentrations reported for the same NHANES participants [Centers for Disease Control and Prevention (CDC) 2005]. Moreover, although the DEHP metabolites are excreted in the urine mainly as glucuronides (Dirven et al. 1993; Peck and Albro 1982), in the ALSPAC pooled sample the urinary concentrations of free and total species of MEHP were essentially equal; for MEHHP and MEOHP, the fractions excreted as a free species were within the expected ranges (Kato et al. 2004). Of note, MEHP can also be formed from DEHP by both biotic and abiotic processes (Koch et al. 2006; Nakamiya et al. 2005; Staples et al. 1997); therefore, MEHP is itself an environmental contaminant. By contrast, no environmental sources of DEHP oxidative metabolites are known (Koch et al. 2006). These results suggest that the MEHP concentrations measured in this pooled sample were likely the result of contamination with DEHP or MEHP during or after collection (Holmes et al., in press). Therefore, this MEHP concentration, although analytically valid, should not be used for exposure or risk assessment purposes. In addition to the higher potential for external contamination of MEHP compared with the oxidative metabolites, MEHP has a shorter elimination half-life and represents a smaller fraction of the DEHP urinary metabolites. Together, these results suggest that MEHP is a weaker biomarker of exposure to DEHP than the DEHP oxidative metabolites (Koch et al. 2006). Therefore, using only MEHP for exposure or risk assessment—particularly in archived biological samples, where external DEHP or MEHP contamination cannot be excluded—should be avoided.

## Biomonitoring and Field Blanks

Sophisticated analytical chemistry techniques, highly trained laboratory personnel, and strict quality control/quality assurance laboratory practices define high-quality biomonitoring data (Angerer et al. 2007; Caudill et al. 2008; National Research Council 2006; Needham et al. 2007). Other factors, such as the integrity of the specimen, are important to ensure the validity of biomonitoring results (National Research Council 2006).

In recent decades, biomonitoring initiatives have been implemented worldwide, either in support of epidemiologic investigations or as part of national health surveys (CDC 2003; National Children’s Study 2009; Viso et al. 2009). Combining environmental monitoring (e.g., air, water) and exposure history/questionnaire data may also be used to assess human exposure to environmental chemicals and is common in occupational settings (NIOSH 1994). In these scenarios, the collection and storage of the environmental specimens follow strict protocols to guarantee the validity and comparability of the results. In addition to collecting field blank and replicate samples, these protocols often require screening of collection materials to ensure that they do not contain detectable levels of the target chemical (NIOSH 1994).

Environmental chemicals are present in human biological tissues at concentrations considerably lower than in the environment. Because some of these chemicals are rather ubiquitous in the environment, the potential for contamination of the biological specimen during sampling exists. Biomonitoring sampling protocols generally include screening of the collection materials for potential contamination, but they do not routinely include other provisions required for environmental sampling (e.g., field blanks). Commercially available high-purity solvents (e.g., water, methanol) placed in a sample container and processed as a specimen could serve as field blanks. Therefore, incorporating field blanks into biomonitoring programs should not be difficult. In addition to providing a control for evaluating contamination during processing and storage before analysis, field blanks would be useful in determining whether archived specimens could be analyzed for a given chemical, even though the sampling materials may have not been prescreened for the presence of such a chemical. Therefore, we strongly advocate including field blanks in all ongoing and future biomonitoring initiatives. Nonetheless, although having field blanks would strengthen all biomonitoring programs, the absence of field blanks does not necessarily invalidate these programs’ results.

## Collection of Biological Specimens in Medical Settings or after Medical Interventions

For several chemicals (e.g., mercury, DEHP, BPA), acute exposure can occur as a result of medical interventions [Barregard et al. 1995; Calafat et al. 2004, 2009; Food and Drug Administration (FDA) 2001; Koch et al. 2005a]. However, many sources of exposure to such chemicals, especially those that are not considered “active” in a given product, are unknown. The following example illustrates the potential impact on interpreting biomonitoring results when the biological specimens are collected in medical settings. More important, this example highlights the need for additional research to identify all sources and pathways of human exposure to these chemicals, particularly for those used extensively and suspected to affect human health.

One hundred fifty pregnant women participating in a prospective study of pesticides and other endocrine disruptors in maternal and fetal compartments were put on intravenous injection for glucose, water, and electrolyte balance support upon arrival at a hospital for a scheduled cesarean birth. Maternal urine specimens, collected before delivery but after a Foley tube and the intravenous line were placed, were analyzed for phthalate metabolites (Yan et al. 2009). Among these women, the urinary concentrations of most metabolites were similar to or lower than those among the U.S. general population from NHANES 2001–2002 (CDC 2005). However, the median urinary concentrations of the DEHP oxidative metabolites MEHHP (108.9 μg/L) and MEOHP (95.1 μg/L) were more than 5 times their corresponding NHANES concentrations; for MEHP, the median (114.7 μg/L) was more than 20 times higher. DEHP, approved by the FDA for medical uses (FDA 2001), is a plasticizer in PVC plastics, which can be used in medical tubing and blood storage bags. Therefore, the higher-than-population-based urinary concentrations of DEHP metabolites among these women likely reflect their exposures to DEHP in the hospital. This example further illustrates the limitations of MEHP as exposure biomarker because the collection of the urine (directly in a cup or through the Foley tube into a bag) could affect the MEHP urinary concentrations, because DEHP/ MEHP may leach from some of these materials, as well as from the intravenous line. In contrast, the concentrations of the oxidative metabolites, which cannot be formed except through enzymatic processes, would reflect these women’s acute DEHP exposure, thus confirming the validity of the DEHP oxidative metabolites as exposure biomarkers (Koch et al. 2006).

This example also emphasizes that biomonitoring for chemicals that are widely used in consumer and personal care products (e.g., phthalates, BPA, parabens) requires additional considerations beyond choosing optimal exposure biomarkers and analytical methods. Even if sampling materials are pre-screened and known to be contaminant-free, and field blanks are collected, the study design itself, specifically the timing and mode of collecting the biological samples, may involve the use of materials or products that contain the target compounds (or their precursors) (National Toxicology Program 2008). In the example above, the concentrations of the DEHP metabolites, although accurate and reflective of a real exposure to DEHP at the time of delivery, cannot be used as surrogates for DEHP exposure throughout gestation or even for exposures of the general population.

## Representativeness of the Biological Specimen

Biological samples are complex in nature. Because some may be difficult to obtain and may be available only in small amounts, rigorous protocols for collecting these samples are needed. In addition, it is crucial that the specimens used for biomonitoring truly reflect the composition of the original sample. Maintaining a sample’s representativeness starts when separating samples into specimens, generally performed to reduce repeated thaw/freeze cycles, thus minimizing potential contamination and degradation (National Research Council 2006). The sample must be fully thawed (if frozen before) and mixed before making aliquots, and care must be taken to ensure the correct labeling of each specimen.

Further, the concept of representativeness may be of particular interest in the case of samples collected from infants, young children, and pregnant women and in situations where cross-contamination of the specimen with other tissues/fluids can occur. For example, urine collected from a woman during her period could be tainted with blood, and contamination of seminal fluid with urine cannot be ruled out. In these situations, we recommend that the potential for contamination be noted. Furthermore, guidance for the collection of samples to minimize potential cross-matrix contamination in such situations is needed. Amniotic fluid, cord blood, and meconium are promising matrices for assessing prenatal exposures, a period when humans are highly susceptible to potential adverse health effects from exposure to certain chemicals. If cross-contamination of the specimen occurs, biomarkers measured in meconium and in amniotic fluid would reflect exposure not only during gestation but also during the neonatal period or during delivery, particularly for ubiquitous chemicals or those commonly present in medical settings. Therefore, it is critical that the personnel responsible for collecting the samples appropriately document all events related to the collection and communicate them to the study principal investigator and the analytical laboratory personnel.

Cross-contamination of amniotic fluid with the mother’s blood during delivery might affect primarily the amniotic fluid concentrations of persistent chemicals that are normally measured in blood or serum/plasma. By contrast, cross-contamination of meconium or another matrix with urine would likely have a bigger impact for nonpersistent chemicals, which are metabolized and eliminated primarily in the urine, than for persistent chemicals that undergo rather limited urinary excretion. To minimize the potential impact of cross-contamination of meconium/feces with urine, additional measures for standardizing the collection procedures, such as avoiding the use of diapers containing meconium/feces that also appear to be wet, can be implemented (Calafat and Needham 2008). Although measuring chemicals in complex biological matrices is analytically possible (Needham et al. 2005), because of potential uncertainties during collection, interpreting the concentrations of biomarkers in matrices with relatively high potential for cross-contamination should be conducted cautiously.

One other consideration that may affect the representativeness of a given sample relates to the collection of urine by using absorbent materials (e.g., diapers). First, one must ensure that these materials do not contain the target chemical. Second, unlike urine collected directly in a urine cup, bag, or similar container, these specimens need to be extracted from the absorbent material before their analyses (Lee and Arbuckle 2009). As expected, the urine, other urinary biomolecules or solutes, and both conjugated and free urinary species of the target chemicals will be only partially recovered, and the composition of the extracted urine will change. The extraction efficiency of a given compound relates to its aqueous solubility, which strongly depends on its chemical structure—which determines its physicochemical properties (e.g., lipophilicity, ionizability)—and on the nature of the solution (i.e., urine), which is affected by pH, ionic strength, temperature, and other solutes (Kerns et al. 2008).

In general, the recovery from absorbent materials of the urinary conjugates of a chemical will be higher than that of the less hydrophilic free species. Because organic chemicals are excreted mostly as urinary conjugates, interpretation of biomonitoring results should not be affected considerably provided an adequate extraction of the conjugates exists. Nonetheless, because of the differential extraction losses, exposures to organic chemicals, if estimated from the concentrations of free and conjugated (i.e., total) species in urine collected from absorbent materials, may be somewhat underestimated, whereas the fraction of the chemicals excreted as conjugates may be overestimated. Therefore, urine sampling methods from infants and young children (Lee and Arbuckle 2009) should be examined for their potential impact in the exposure assessment process. Other methods that do not require using absorbent materials should be evaluated.

## Recommendations for Best Biomonitoring Practices

Adequate generation of biomonitoring data requires validated and high-quality analytical methods, qualified laboratory personnel, and strict quality control/quality assurance laboratory practices. Other important aspects include the selection of the most relevant bio-markers and understanding of the potential effects of the biological matrix on the bio-markers’ concentrations. Furthermore, other factors, including adequate collection, handling, shipping, and storage procedures to preserve the integrity of the specimen and the target analytes, must be considered to guarantee the valid interpretation of the biomonitoring data, particularly for chemicals with widespread commercial and industrial use.

Recommendations for sampling and processing approaches applicable to ongoing and future biomonitoring initiatives include the following:

- Inclusion of field blank samples (e.g., high-purity solvent(s) placed in a sample container and processed as a biological specimen) in the protocols for the collection and/or processing of biological specimens for all programs/studies with a current or potential biomonitoring component.
- To the fullest extent possible, evaluation *a priori* of the potential impact of the collection setting on the biomonitoring concentrations (especially of chemicals that may be present in commonly used products). Available data suggest that the main issues relate to collecting biological samples from pregnant women at the time of delivery (e.g., use of intravenous line) or from persons undergoing intensive care and/or outpatient medical treatment (e.g., platelet donation, dialysis). Therefore, additional research is needed to identify all sources and pathways of human exposure to these widely used chemicals, many of which are not considered “active” ingredients in commercial products.
- Recording of the parameters related to collecting and processing of samples. This includes information on the sampling time and location (e.g., home, hospital, work-place), whether sampling was embedded into prescheduled or ad hoc health visits (e.g., child well-being, prenatal care appointments, amniocentesis, delivery), and detailed description of collection procedures (e.g., urine collected in a cup or diaper, or through a catheter) and of the processing (e.g., making aliquots, storage and shipping conditions) of the samples before arrival to the laboratory for analysis.
- Evaluation of the sampling collection protocols to identify the potential for cross-matrix contamination (e.g., urine or amniotic fluid with blood, meconium/feces, or seminal fluid with urine).
- Consideration of the extraction efficiency of urinary species of chemicals from diapers when interpreting biomonitoring data from infants and young children. It is possible that the conjugation capability, assessed from the percentage of conjugated species, would be somewhat overestimated. By contrast, exposure, categorized from the urinary concentrations of the total (free plus conjugated) species, would be underestimated.
- Examination of the methods for collecting urine from infants and young children for their potential impact on the exposure assessment process (e.g., changes in the composition of urine extracted from a diaper), and evaluation of collection methods not relying on the use of absorbent materials for their applicability in biomonitoring studies.

Biomonitoring requires a team approach. Therefore, it is critical to facilitate constructive dialog and partnership among laboratory and field researchers and study participants from the onset of the study to ensure its success.
